# Middle East respiratory syndrome coronavirus (MERS-Cov) screening of exposed healthcare workers in a tertiary care hospital in Saudi Arabia

**DOI:** 10.1186/2047-2994-4-S1-O57

**Published:** 2015-06-16

**Authors:** K Alameer, B Abukhzam, W Khan, A El-Saed, H Balkhy

**Affiliations:** 1 Infection Prevention and Control, Riyadh, Saudi Arabia; 2 King Abdulaziz Medical City, Riyadh, Saudi Arabia

## Introduction

The possibility of healthcare exposure to Middle East Respiratory Syndrome Coronavirus (MERS-Cov) has been early described. Almost one-third of the confirmed MERS-Cov cases in Saudi Arabia were among healthcare workers (HCWs).

## Objectives

To describe three-season experience of MERS-Cov exposure and outcome among HCWs in a tertiary care hospital in Saudi Arabia.

## Methods

Prospective surveillance was conducted in King Abdulaziz Medical City in Riyadh, Saudi Arabia for unprotected exposed HCWs, with every newly PCR-confirmed MERS-Cov case, between June 2013 and March 2015. HCWs exposed to confirmed MERS-Cov patients were examined for the presence of symptoms and nasopharyngeal (and rarely other) swab was obtained for MERS-Cov. Exposure was defined as caring of or being in close proximity (within 2 meters) of a confirmed patient, without proper personal protective equipment (PPE).

## Results

During the duration covered, a total 32 patients with PCR-confirmed MERS-Cov were associated with exposure of 1361 HCWs. Only 328 (24.1%) of the exposed HCWs had symptoms suggestive of respiratory infection at the time of screening. MERS-Cov was confirmed in only 14 (1.03%) HCWs. MERS-Cov confirmation was roughly similar among symptomatic (3/328, 0.91%) and asymptomatic (11/1033, 1.06%) HCWs. Only 2 (14.3%) of the 14 confirmed HCWs required hospitalization. While the mortality among the confirmed patients was very high (21/32, 65.6%), none of the 14 confirmed healthcare workers died.

## Conclusion

We are reporting high potential of healthcare exposure to MERS-Cov in the healthcare setting but a very low transmission rate. Proper compliance with PPEs is essential to further reduce unprotected exposure of the HCWS. In essence contact tracing and testing for all unprotected exposed HCWs to MERS CoV irrespective of symptoms remain critical measures to prevent or reduce the impact of MERS-Cov hospital outbreak till further understanding of MERS-Cov behavior is delineated.

## Disclosure of interest

None declared.

